# Angiotensin‐converting enzyme 2 (ACE2), SARS‐CoV‐2 and the pathophysiology of coronavirus disease 2019 (COVID‐19)

**DOI:** 10.1002/path.5471

**Published:** 2020-06-10

**Authors:** Arno R Bourgonje, Amaal E Abdulle, Wim Timens, Jan‐Luuk Hillebrands, Gerjan J Navis, Sanne J Gordijn, Marieke C Bolling, Gerard Dijkstra, Adriaan A Voors, Albert DME Osterhaus, Peter HJ van der Voort, Douwe J Mulder, Harry van Goor

**Affiliations:** ^1^ Department of Gastroenterology and Hepatology, University Medical Center Groningen University of Groningen Groningen The Netherlands; ^2^ Department of Internal Medicine, Division of Vascular Medicine, University Medical Center Groningen University of Groningen Groningen The Netherlands; ^3^ Department of Pathology and Medical Biology, University of Groningen University Medical Center Groningen Groningen The Netherlands; ^4^ Department of Internal Medicine, Division of Nephrology, University Medical Center Groningen University of Groningen Groningen The Netherlands; ^5^ Department of Obstetrics and Gynecology, University Medical Center Groningen University of Groningen Groningen The Netherlands; ^6^ Department of Dermatology, University Medical Center Groningen University of Groningen Groningen The Netherlands; ^7^ Department of Cardiology, University Medical Center Groningen University of Groningen Groningen The Netherlands; ^8^ Research Center for Emerging Infections and Zoonoses University of Veterinary Medicine Hannover Germany; ^9^ Department of Critical Care Medicine, University of Groningen University Medical Center Groningen Groningen The Netherlands

**Keywords:** angiotensin‐converting enzyme 2 (ACE2), coronavirus disease 2019 (COVID‐19), severe acute respiratory syndrome coronavirus 2 (SARS‐CoV‐2), renin–angiotensin–aldosterone system (RAAS), pathophysiology, pathology, organ involvement, risk factors, treatment

## Abstract

Angiotensin‐converting enzyme 2 (ACE2) has been established as the functional host receptor for severe acute respiratory syndrome coronavirus 2 (SARS‐CoV‐2), the virus responsible for the current devastating worldwide pandemic of coronavirus disease 2019 (COVID‐19). ACE2 is abundantly expressed in a variety of cells residing in many different human organs. In human physiology, ACE2 is a pivotal counter‐regulatory enzyme to ACE by the breakdown of angiotensin II, the central player in the renin–angiotensin–aldosterone system (RAAS) and the main substrate of ACE2. Many factors have been associated with both altered ACE2 expression and COVID‐19 severity and progression, including age, sex, ethnicity, medication, and several co‐morbidities, such as cardiovascular disease and metabolic syndrome. Although ACE2 is widely distributed in various human tissues and many of its determinants have been well recognised, ACE2‐expressing organs do not equally participate in COVID‐19 pathophysiology, implying that other mechanisms are involved in orchestrating cellular infection resulting in tissue damage. Reports of pathologic findings in tissue specimens of COVID‐19 patients are rapidly emerging and confirm the established role of ACE2 expression and activity in disease pathogenesis. Identifying pathologic changes caused by SARS‐CoV‐2 infection is crucially important as it has major implications for understanding COVID‐19 pathophysiology and the development of evidence‐based treatment strategies. Currently, many interventional strategies are being explored in ongoing clinical trials, encompassing many drug classes and strategies, including antiviral drugs, biological response modifiers, and RAAS inhibitors. Ultimately, prevention is key to combat COVID‐19 and appropriate measures are being taken accordingly, including development of effective vaccines. In this review, we describe the role of ACE2 in COVID‐19 pathophysiology, including factors influencing ACE2 expression and activity in relation to COVID‐19 severity. In addition, we discuss the relevant pathological changes resulting from SARS‐CoV‐2 infection. Finally, we highlight a selection of potential treatment modalities for COVID‐19. © 2020 The Authors. *The Journal of Pathology* published by John Wiley & Sons Ltd on behalf of Pathological Society of Great Britain and Ireland.

## Introduction

Coronavirus disease 2019 (COVID‐19) is caused by the recently emerged coronavirus, SARS‐CoV‐2, which was first reported in December 2019 in the city of Wuhan, Hubei province, PR China [1]. Similar to other coronaviruses (SARS‐CoV‐1 and MERS‐CoV), human‐to‐human transmission is well established for this virus, which has now spread globally [1, 2]. The World Health Organization (WHO) has estimated the expected number of secondary cases for each infected individual (basic reproduction number, *R*_0_) to range from 2.0 to 2.5, although this number is gradually decreasing upon the implementation of epidemiological management strategies [3].

Identically to SARS‐CoV‐1, which was responsible for the SARS outbreak in 2002–2004, the main target of SARS‐CoV‐2 is the respiratory tract, leading to typical clinical signs including fever, dry cough, fatigue, and dyspnoea [4]. Typically, the disease progresses to a severe form in 10–20% of patients, requiring hospital admission or even intensive care unit (ICU) treatment [5]. Characteristic laboratory features include lymphopenia, elevated levels of C‐reactive protein (CRP), lactate dehydrogenase (LDH), and aspartate aminotransferase (AST) [4]. Risk factors for an unfavourable outcome include older age, male gender, high body‐mass index (BMI), and underlying comorbidities such as obesity, hypertension, cardiovascular disease, diabetes, or chronic respiratory disease [6]. Current clinical management strategies include prevention of further dissemination of the virus, control of inflammation, and supportive care, the latter intended to maintain efficient respiratory gas exchange through oxygen supplementation, positive airway pressure, and mechanical ventilation. Effective and safe disease‐modifying or preventive treatments, such as targeted antiviral drugs, biological response modifiers or vaccines, are not yet available.

**Figure 1:**
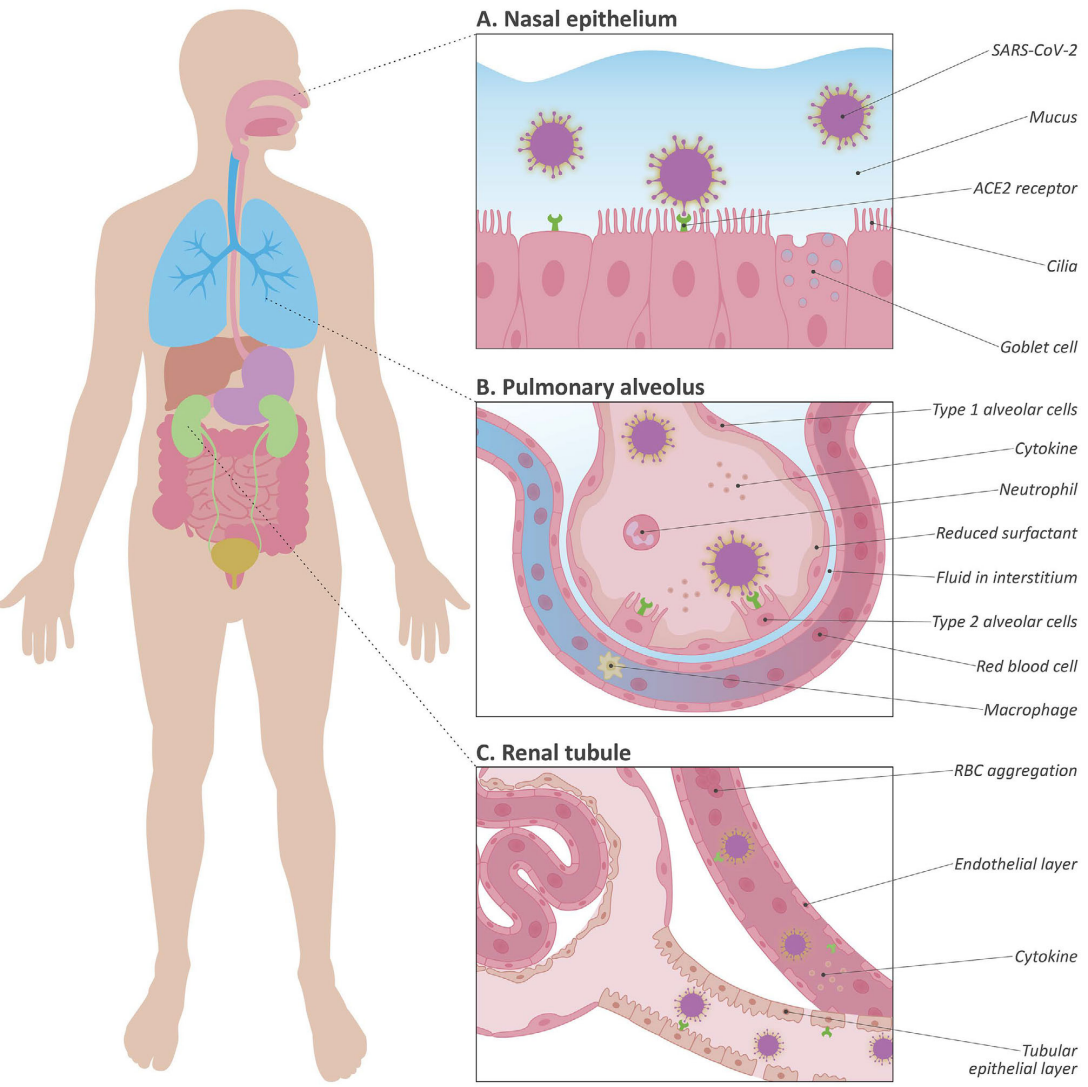
(A–C) Simplified representation of SARS‐CoV‐2 infection and the role of ACE2 in this process. (A) First, SARS‐CoV‐2 may pass through either the mucous membranes, primarily the nasal epithelia, by binding to the ACE2 receptor. (B) In addition, SARS‐CoV‐2 can directly enter the respiratory tract and infect respiratory epithelial cells. After infection, extensive diffuse alveolar damage occurs in the lungs, followed by bilateral oedema, diffuse reactive hyperplasia of type II pneumocytes, thickening of alveolar septa, and infiltration of inflammatory cells. (C) A simplified representation of COVID‐19‐related renal involvement. Typical COVID‐19‐associated changes in the kidneys are diffuse tubular injury with loss of brush border integrity, endothelial damage of the capillaries, and erythrocyte aggregates occluding the capillary lumina.

Angiotensin‐converting enzyme 2 (ACE2), the functional receptor of SARS‐CoV‐2, plays a crucial role in the pathogenesis of COVID‐19, as it provides viral entry into human cells [7, 8]. The viral spike (S) protein of SARS‐CoV‐2 binds to ACE2 as a cellular receptor in a similar way to SARS‐CoV‐1. Importantly, SARS‐CoV‐2 is more pathogenic, at least in part because of its 10‐ to 20‐fold increased binding affinity to ACE2 [8, 9]. This binding leads to host cell entry of the virus in concert with S‐protein priming by the host cell protease TMPRSS2. Evidently, SARS‐CoV‐2 cell entry and pathologic effects mainly occur in cells of the (upper) respiratory tract [10, 11]. Further dissemination in the host, such as in the kidneys or the gastrointestinal tract, may be related to local ACE2 expression (Figure 1). Since identifying the exact role of ACE2 and SARS‐CoV‐2 in COVID‐19 may have major implications for understanding the disease, we reviewed their involvement in the pathogenesis of organ damage in COVID‐19. Furthermore, we highlight a selection of currently considered treatment modalities for COVID‐19.

## Angiotensin‐converting enzyme 2 (ACE2)

### ACE2 in human physiology

**Figure 2:**
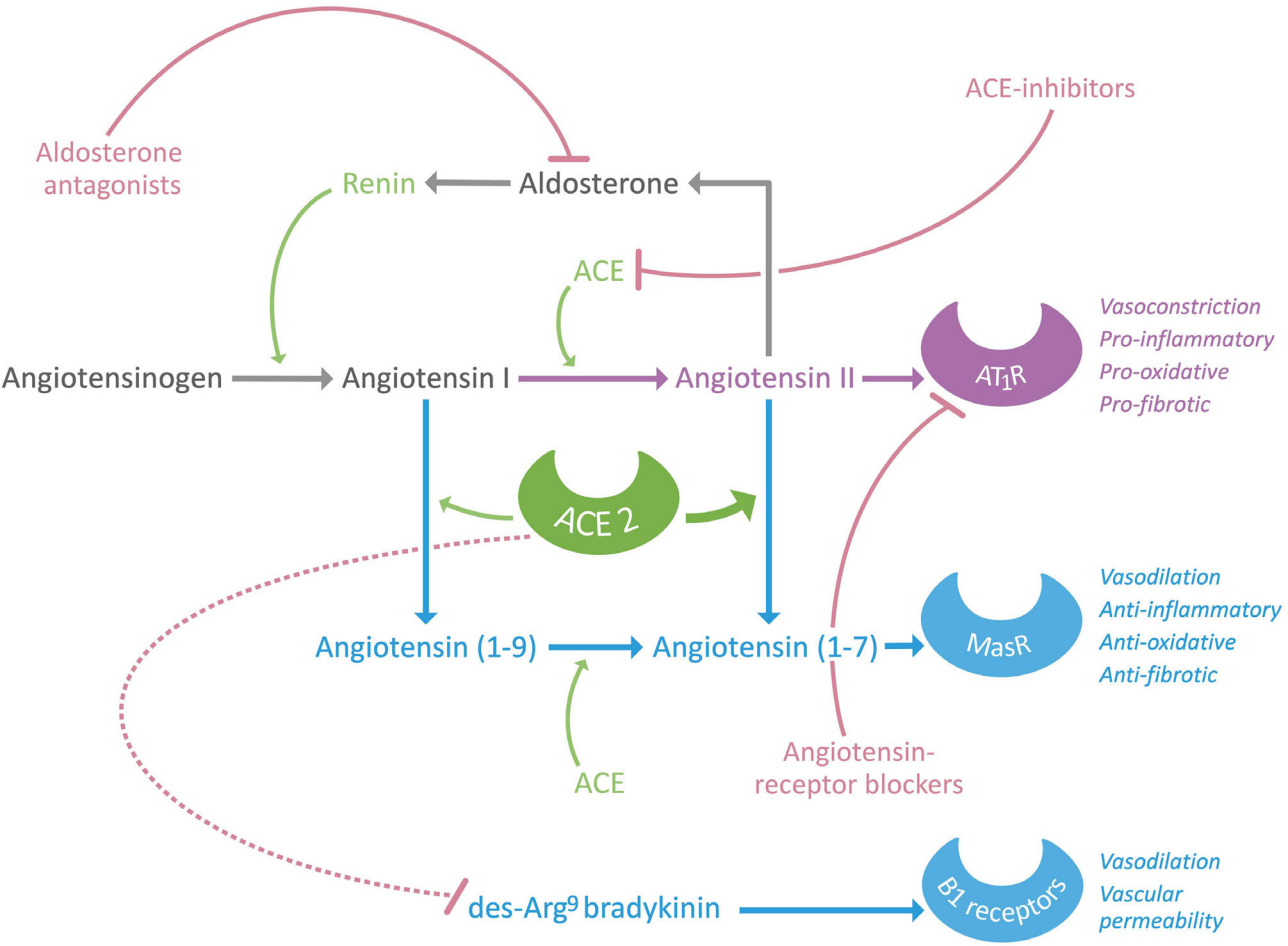
Structure of the renin‐angiotensin‐aldosterone system (RAAS), the role of ACE2 in this physiological system, and potential treatment targets.

ACE2 is a homologue of angiotensin‐converting enzyme (ACE) and plays a pivotal role in the renin–angiotensin–aldosterone system (RAAS), involving blood pressure regulation and electrolyte homeostasis (Figure 2). Angiotensinogen, produced by the liver, is cleaved by renin, resulting in the formation of angiotensin I (Ang I). Subsequently, ACE is one of the enzymes that catalyses the conversion of Ang I to angiotensin II (Ang II) [12]. Ang II, the main active RAAS component, exerts its effects mainly via angiotensin II type 1 receptors (AT_1_R). Major effects of Ang II include vasoconstriction, renal sodium reabsorption and potassium excretion, aldosterone synthesis, blood pressure elevation, and induction of inflammatory and pro‐fibrotic pathways [13, 14]. ACE2 cleaves Ang II to angiotensin(1–7), which exerts vasodilating, anti‐inflammatory, and anti‐fibrotic effects through binding to the Mas receptor [15]. In addition, ACE2 cleaves Ang I into angiotensin(1–9), which is in turn converted into angiotensin(1–7) by ACE, although this mechanism is usually of less physiological importance [16]. Therefore, ACE2 functionally counteracts the physiological role of ACE, and the eventual effects of RAAS activation depend on the tissue ACE/ACE2 balance, which determines the availability of different angiotensin peptides and hence the balance between pro‐inflammatory and pro‐fibrotic, and anti‐inflammatory and anti‐fibrotic pathways [16]. This balance can be affected by many factors, including pharmacological RAAS blockade in several disease conditions. Furthermore, several dietary risk factors for cardiometabolic disorders such as high sodium intake, high fat intake, and high fructose intake shift the ACE/ACE2 balance towards pro‐inflammatory and pro‐fibrotic (ACE‐mediated) effects [17, 18, 19, 20].

Apart from its functions in RAAS, ACE2 orchestrates bradykinin metabolism in the lungs by inactivating des‐Arg^9^ bradykinin, which is a potent ligand of bradykinin receptor type 1 (B1), thereby inhibiting effects such as vasodilation and elevation of vascular permeability [21]. In the gastrointestinal tract, ACE2 has been described as a key regulator of dietary amino acid homeostasis, expression of antimicrobial peptides, local innate immunity, and gut microbial ecology. In fact, transplantation of gut microbiota from *Ace2*‐knockout mice resulted in an increased propensity to develop severe colitis [22].

### Tissue distribution of ACE2

Previously, we investigated the immunolocalisation of ACE2 in healthy human organs [7]. ACE2 was highly expressed on lung alveolar epithelial cells and small intestinal epithelial cells, consistent with potential routes of viral transmission of SARS‐CoV‐2, as both respiratory and gastrointestinal systems share interfaces with the external environment. Additionally, ACE2 was present on vascular endothelial cells and smooth muscle cells in all organs studied. In the kidney, ACE2 was strongly expressed in the brush border of proximal tubular cells and moderately or weakly in parietal epithelial cells and podocytes, whereas ACE2 staining was weak or negative in glomerular endothelial cells and mesangial cells. ACE2 was also present in the basal epidermal layer of the skin and in the oral and nasal mucosa. In contrast, ACE2 was absent in lymphoid tissues and hepatobiliary structures [7]. The intense staining on various epithelial cells (small intestine, kidney, skin) strongly suggests RAAS‐independent functions of ACE2. These findings trigger alternative hypotheses regarding ACE2 involvement in viral transmission pathways. Furthermore, we previously noted that endothelial ACE2 was upregulated in the glomerular and interstitial capillaries in kidney diseases independent of the initial trigger, indicating that ACE2 may also be viewed as a damage marker [23]. Summarising, ACE2 is widely expressed in human tissues, both in principal target organs of SARS‐CoV‐2 and in organs that play a seemingly less important or even unknown role in COVID‐19 pathophysiology.

### Interaction between ACE2 and SARS‐CoV‐2

**Figure 3:**
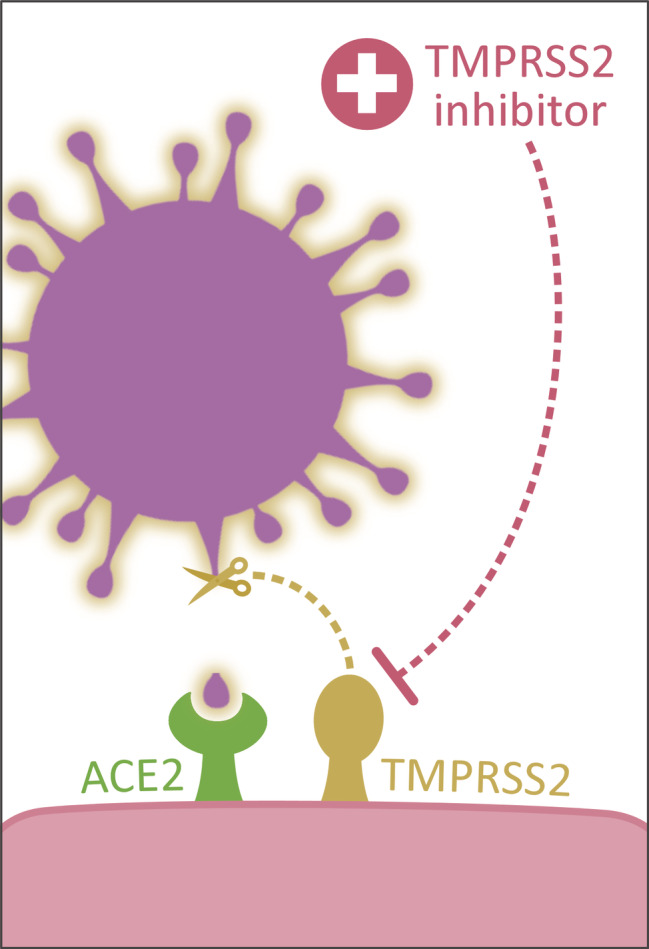
SARS‐CoV‐2 interacts with ACE2 as host cell receptor. In addition to binding, priming of the viral spike (S) protein by the host serine protease TMPRSS2 is required for cell entry.

Recently, ACE2 was unequivocally established as the functional host receptor for SARS‐CoV‐2 (Figure 3) [8]. Binding kinetics revealed a 10‐ to 20‐fold higher binding affinity compared with the SARS‐CoV‐1 virus [8, 9]. These findings may partially explain the apparently easier transmissibility of SARS‐CoV‐2 and that increased ACE2 expression may confer increased susceptibility to host cell entry of SARS‐CoV‐2. It was previously shown that a specific region within the SARS‐CoV‐1 spike protein interacts with ACE2, leading to fusion with the host cell membrane [16, 24]. An experimental animal study in *Ace2*‐knockout mice further underlined the importance of this receptor in the pathogenesis of SARS caused by SARS‐CoV‐1 [25]. The authors hypothesised that infection with SARS‐CoV‐1 results in ACE2 downregulation through its internalisation, induced by binding of SARS‐CoV‐1 to ACE2, as a mechanism contributing to the severity of lung pathologies [25]. Consequently, this would lead to impairment of the protective effect of ACE2 on the severity of acute respiratory distress syndrome (ARDS). This, as well as a harmful effect of Ang II, was previously demonstrated in several animal models of ARDS [26, 27, 28, 29]. The interaction between ACE2 and SARS‐CoV‐1 and with SARS‐CoV‐2, and further downstream effects, exhibit a high level of similarity between each other [8]. During hypoxia, Ang II‐induced pulmonary vasoconstriction occurs, aimed to restore the ventilation–perfusion mismatch, but simultaneously inducing adverse pro‐fibrotic effects, which both are ameliorated by concomitant upregulation of ACE2 [30]. Under similar circumstances, SARS‐CoV‐2‐induced downregulation of ACE2 could impair clearance of Ang II and hence lead to aggravation of tissue damage. On the other hand, one may speculate that ACE2 downregulation by SARS‐CoV‐2 results in a decreased opportunity for further viral cell entry, thereby limiting viral spread. However, as one may hypothesise that SARS‐CoV‐2 infects ACE2‐expressing cells with greater efficiency compared with SARS‐CoV‐1, presumably through exploiting cellular factors promoting viral attachment and entry, it is likely that SARS‐CoV‐2 viruses would need less ACE2 to enable viral spread. Taken together, the role of ACE2 in SARS‐CoV‐2 cellular infection is complex and not yet defined, which makes it interesting to study if and how SARS‐CoV‐2 interferes with ACE2 expression and/or its regulation, and how this influences viral replication.

## Risk factors for COVID‐19 severity and ACE2 expression

### Genetic factors

ACE2 is encoded by a gene located on chromosome Xp22 and consists of at least 18 exons and 20 introns, amounting to approximately 40 kb of genomic DNA [31]. The genetic architecture closely resembles the structure of the *ACE* gene and may lead to a variety of alternative RNA transcripts. The *ACE2* gene is characterised by a number of polymorphisms, which have been associated with the diversity of RAAS‐system pathologies, such as essential hypertension [32]. However, the genetic background of ACE2 expression and functionality across different populations in relation to SARS‐CoV‐2 is largely unknown. Comparative systematic analysis of coding‐region variants and expression quantitative trait loci (eQTL) variants of ACE2 across different populations showed higher allele frequencies of eQTL variants associated with higher ACE2 tissue expression levels in East Asian compared with European populations. This may imply a differential susceptibility to SARS‐CoV‐2 infection across different populations. However, no evidence supporting potential S‐protein binding‐resistant ACE2 mutants was obtained [33]. Structural modelling and superimposition analyses of the native ACE2‐ and ACE2‐S‐protein complex were used to study changes in ACE2 variants and the intermolecular interactions with the S‐protein. Most ACE2 coding variants showed high structural similarity and highly similar binding affinity with the S‐protein of SARS‐CoV‐2. However, two allelic variants were identified that demonstrated considerable variation in intermolecular interaction with the S‐protein, showing varying spatial orientation of key interacting residues of ACE2 [34]. These ACE2 genetic variations may provide a basis for relative or complete potential resistance against SARS‐CoV‐2 infection.

### Age and sex

ACE2 expression in the lungs and SARS‐CoV‐2 viral load have been suggested to increase with age, which might provide an explanation for the higher disease severity observed in older patients with COVID‐19 [35]. Advancing age is increasingly recognised as one of the strongest predictors for severe COVID‐19 [6]. Older adults (aged above 60 years) are at increasing risk of contracting severe COVID‐19 with higher complication and case fatality rates [36]. Similar to influenza and other respiratory viral infections, gradually decreasing innate and adaptive immune responses may be expected to play an important role in this age‐related increased susceptibility. Accumulating data also show the existence of a gender‐associated predisposition to COVID‐19, with men being more prone to develop severe disease than women [37]. ACE2 expression may be a contributing factor to this association as single‐cell transcriptomics demonstrated that ACE2 expression was higher among Asian men than Asian women [38]. Observational data indicated higher frequencies of males among critically ill patients [39, 40]. In line, males appeared to be more frequent among deceased patients compared with recovered patients [41]. Possible explanations of male predominance among COVID‐19 patients may be differences in exposure, smoking behaviour, other lifestyle factors, differences in chromosomal ACE2 expression, ACE2 expression in testicular tissue, sex hormone‐driven immune system regulation, or gender differences in RAAS regulation [37, 42, 43, 44]. Interestingly, in two independent cohorts of patients with heart failure, plasma concentrations of ACE2 were higher in men than in women [45].

### Obesity

Obese patients with COVID‐19 may have an increased risk of ICU admission and mortality. Although obese patients frequently present with mechanical hypoventilation (leading to hypercapnic respiratory failure), those with COVID‐19 present with hypoxic respiratory failure. This led to discussions about a potential role of fat tissue in COVID‐19 pathogenesis in relation to ACE2 expression. Granting that obesity predisposes to developing chronic disease, obesity could also be an independent risk factor for COVID‐19 [46]. BMI is significantly higher among COVID‐19 patients with critical disease requiring ICU admission compared with less severe cases [47]. Likewise, the proportion of patients with BMI > 25 kg/m^2^ was significantly elevated in deceased patients compared with survivors. A Chinese multi‐centre study reported significantly higher BMI values among patients with severe disease compared with patients having only mild disease [48]. In other emerging large case series, obesity remains common and may be a risk factor for respiratory distress, eventually requiring mechanical ventilation [49, 50]. These observations are analogous to other respiratory viral infections, for instance the H1N1 influenza virus infection. During that 2009 pandemic, obesity also emerged as an independent risk factor for hospitalisation and death [51, 52]. This could be attributed to obesity‐induced impairment of the immune response, as has been well documented for H1N1 influenza [53]. Mechanistically, adipose tissue‐derived inflammation in obesity leads to substantial metabolic disturbances that could eventually lead to complications such as dyslipidaemia, hypertension, diabetes, cardiovascular disease (metabolic syndrome or Syndrome X), and chronic respiratory failure [54]. Visceral fat tissue can induce pro‐inflammatory effects, which are regulated by adipokines and Ang II. Interestingly, ACE2 is abundantly present on visceral adipocytes [7, 55]. ACE2 on adipocytes exerts systemic effects on the cardiovascular system and experimental studies demonstrated interactions between gender, adipocyte ACE2, and complications of obesity, e.g. hypertension [56]. Of note, leptin is one of the most important adipokines driving these pro‐inflammatory effects and higher leptin availability has been associated with increased Ang II levels as well as decreased ACE2 expression and activity [57]. In addition, high leptin levels have been associated with accumulation of alveolar fluid and increased inflammation upon hypoxia and ARDS [58]. Therefore, it may be hypothesised that excess visceral adipose tissue in patients with COVID‐19 may drive disease progression – whether or not affected by gender – especially through aggravating the cascade of hyperinflammatory reactions in the disease [59]. Ultimately, this ‘cytokine storm’ may lead to multiple organ failure in patients with COVID‐19.

### Comorbidity

A recent meta‐analysis of 46 248 patients diagnosed with COVID‐19 reported that severe disease was associated with hypertension, chronic respiratory disease, and cardiovascular disease [60]. In another report including over 44 000 patients with confirmed COVID‐19, hypertension, chronic respiratory disease, diabetes mellitus, cardiovascular disease, and cancer emerged as the most common comorbidities [1]. Many of these comorbidities are characterised by either increased or decreased ACE2 expression and/or activity, as well as a shift in ACE/ACE2 balance in both directions. This could be related to underlying conditions and/or to treatment with RAAS inhibitors (discussed in the section ‘Pathogenesis and treatment options for COVID‐19’). However, the relative contribution of each of these underlying conditions to disease severity and mortality remains undetermined. Many of the currently available reports were unadjusted for potential confounding factors, including age, sex, and lifestyle factors such as smoking and diet. Similarly, many studies were uncontrolled, had relatively short follow‐up periods, or were likely affected by inaccurate scoring or under‐diagnosis [61].

### Immunosuppressive drugs

In general, it is advised that patients using immunosuppressive drugs should not pre‐emptively stop their medication, because there is still much unknown about potential risks or benefits. For instance, transplanted patients frequently use ciclosporin, which has been shown to have antiviral activity against SARS‐CoV‐1 [62]. Patients with chronic immune‐mediated inflammatory diseases [IMIDs, e.g. rheumatoid arthritis (RA) or inflammatory bowel diseases (IBD)] who are treated with cytokine inhibitors (e.g. TNF antagonists, anti‐IL6R therapy) do not seem to be at an automatically increased risk of developing severe COVID‐19 [63]. Although at first sight these treatments may seem to lead to immune suppression and may therefore be considered potentially harmful in the context of COVID‐19, they specifically target individual inflammatory cytokines or mediators instead of a broad panel of immune system components. In fact, cytokine inhibitors potentially attenuate the hyperinflammatory state associated with COVID‐19 and may therefore exert beneficial effects. This concept is supported by observations that the pro‐inflammatory cytokines induced in COVID‐19 seem to be more crucial for the host inflammatory response compared with those involved mainly in viral clearance [63]. Patients with solid malignancies treated with immune checkpoint inhibitors (ICIs), including anti‐programmed death (‐ligand)‐1 (PD‐1/PD‐L1), anti‐cytotoxic T‐lymphocyte‐associated protein‐4 (CTLA‐4), and chimeric antigen receptor (CAR) T‐cell therapy for certain B‐cell‐related haematological malignancies, frequently experience T‐cell‐engaging immunomodulatory effects [64]. Well‐known immune‐related adverse events include cytokine release syndrome (CRS) and pneumonitis [65, 66], which in theory might render patients more vulnerable to infections [67]. Interestingly, these complications resemble the clinical presentation of advanced COVID‐19 and respond well to anti‐IL6R therapy [68], providing a strong rationale for anti‐IL6R therapy in COVID‐19 [69].

## Pathological presentation of COVID‐19 and organ involvement

As described, ACE2 expression and activity is ubiquitously present within the human body, but many of its determinants on tissue level dynamics are unknown. However, in COVID‐19 pathophysiology, there is seemingly huge spatiotemporal heterogeneity in organ involvement, presumably because multiple pathophysiological mechanisms may be causally involved in the observed tissue damage. Both SARS‐CoV‐2 infection, directly mediated by ACE2 expression and activity, and superimposed disease triggers may be responsible for the observed pathological findings. Detailed pathological study of tissue specimens is therefore urgently needed to improve our understanding by disentangling the potential origins of tissue damage.

### Respiratory tract involvement

**Figure 4:**
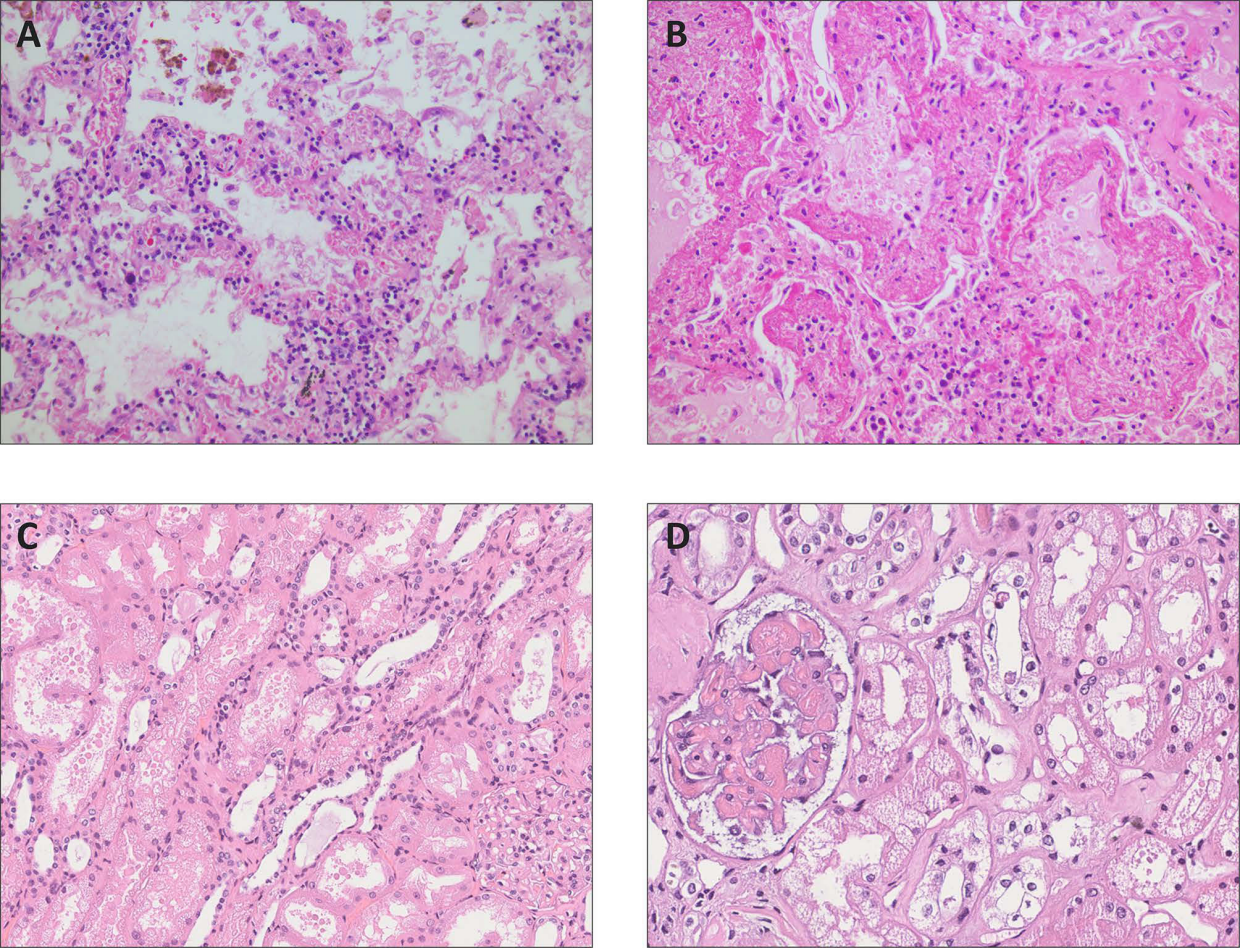
(A–D) Pathological changes in lungs and kidneys from autopsy specimens of patients with COVID‐19. (A) Thickening of alveolar septa with lymphocytic infiltrate and oedema is observed together with damage and release of alveolar epithelial cells and other cellular debris in alveolar spaces. (B) Alveoli with variable thickening of alveolar walls with partial collagen fibrosis (right upper part) and severe damage with, besides cellular debris, intra‐alveolar oedema, protein, fibrin, and hyaline membranes. (C) Pathological changes in kidneys from an autopsy specimen of a patients with COVID‐19. The proximal convoluted tubules show loss of brush border integrity and vacuolar degeneration. This coincides with debris composed of necrotic epithelium in tubular lumina. Erythrocyte aggregates obstructing peritubular capillaries are frequently present. In some cases, inflammatory infiltrates are present in tubules with multiple foci of bacteria and white blood cell casts. (D) Segmental fibrin thrombi were observed in glomeruli, with ischaemic glomerular contraction with the accumulation of leaked plasma in Bowman’s space.

The initial clinical presentation of COVID‐19 consists of respiratory symptoms such as fever, dry cough, shortness of breath, rhinitis, and, additionally, chest pain, myalgia, and/or fatigue [70, 71, 72, 73]. In more severe cases needing hospitalisation, viral pneumonia develops with progressive ground‐glass opacities on chest computed tomography (CT). In clinically critical cases, this is accompanied by further complications including ARDS, cardiac pathology, and secondary infections. Given the similarities between SARS‐CoV‐1 and SARS‐CoV‐2, lung pathology shows considerable equivalence [74, 75]. Hitherto, there are limited reports of mainly autopsy cases describing lung pathological findings [76, 77, 78, 79]. Similar to SARS, COVID‐19‐associated pathological changes in the lungs generally constitute extensive diffuse alveolar damage with bilateral oedema, proteinaceous or fibrin alveolar exudates, and diffuse reactive hyperplasia of type II pneumocytes (Figure 4A,B). In more advanced pathology, hyaline membrane formation was observed with thickened alveolar septa caused by interstitial fibroblast proliferation consistent with fibrosis (Figure 4B). In addition, variable presence of patchy, mainly interstitial infiltration of mononuclear cells has been reported (Figure 4A) and, in some cases, multinucleated giant cells in alveoli with associated viral changes. In contrast to SARS, there is seemingly more thrombo‐embolic pathology observed in specimens from patients with COVID‐19 (discussed in the section ‘Thrombo‐embolic risk’). Also, intra‐alveolar deposition of neutrophilic granulocytes was reported in a few instances, most likely due to superimposed bacterial infection. Another case report showed immunostaining of the Rp3 NP protein from SARS‐CoV‐2, which was prominently expressed on alveolar epithelial cells, as well as in cell debris within the alveolar space [80].

Along the respiratory tract, ACE2 has been observed in nasal and bronchial epithelial cells. In addition, ACE2 is abundantly expressed on the surface of alveolar type II pneumocytes, which also co‐express several other proteins that are involved in the regulation of viral reproduction and transmission, including *TMPRSS2* [38, 81]. Type II pneumocytes usually produce surfactant, maintain their self‐renewal, and exert immunoregulatory functions. Importantly, these cells share the same basement membrane with closely apposed capillary endothelial cells, also expressing high ACE2 levels. These data indicate that type II pneumocytes together with the related capillary endothelium may be a primary site of SARS‐CoV‐2 entrance, resulting in damage to those cells and the alveolo‐capillary membrane and ongoing reactive hyperplasia of type II pneumocytes. As type II pneumocytes remain targets of viral entry and replication, this may lead to a vicious circle of continuing alveolar wall destruction and repair, eventually culminating in progressive severe diffuse alveolar damage. ACE2 upregulation has also been described in airways in patients with chronic respiratory disease who are smokers, which, together with disturbed ciliary movement and abnormal mucus viscosity, may increase disease vulnerability [82]. However, clinical evidence may indicate that smoking does not necessarily lead to increased vulnerability [83]. Recently, it was suggested that the virus could also exploit goblet cells and ciliated cells in the nasal epithelia as entry portals, a plausible primary infection site in many patients [10].

### Cardiovascular involvement

Although COVID‐19 is primarily a severe respiratory illness, acute myocardial injury is frequently observed, manifested by increased levels of high sensitivity cardiac troponin I (cTnI) or cardiac troponin T (cTnT) in up to 28% of laboratory‐confirmed COVID‐19 patients [84, 85]. The presence of myocardial injury was associated with worsened outcome, with 7‐ to 11‐fold increased mortality rates. The highest mortality rates were observed in patients with both elevated TnT levels and pre‐existing cardiovascular disease. Reciprocally, pre‐existing cardiovascular disease predisposes for SARS‐CoV‐2‐induced myocardial injury and COVID‐19‐associated mortality. Whereas the relation between myocardial injury (associated with myocardial infarction, heart failure, and ventricular arrhythmias) and mortality is evident, the aetiology of acute myocardial injury in response to SARS‐CoV‐2 infection is still unresolved. However, several potential mechanisms have been proposed, including SARS‐CoV‐2‐induced myocarditis, cytokine‐mediated injury (i.e. a systemic cardiotoxic cytokine storm), microvascular injury, or stress‐related cardiomyopathy or myocardial infarction [86, 87]. Virus‐induced myocarditis due to infection of cell populations residing in the heart has also been proposed, though this is still unproven [88]. Scattered individual cardiomyocyte necrosis was observed in cardiac tissue from deceased COVID‐19 patients, however without clear signs of myocarditis [88]. Given the critical role of ACE2 for SARS‐CoV‐2 cell entry, resident ACE2‐expressing cell populations in the heart can be potentially infected. Single‐cell RNA sequencing of discarded donor hearts revealed that pericytes, but not cardiomyocytes, express the highest ACE2 levels [89]. This suggests that cardiac pericytes form a potential SARS‐CoV‐2 target cell, which may cause capillary endothelial cell dysfunction upon infection, culminating in myocardial injury. So far, only one case report has been published on the presence of SARS‐CoV‐2 in the heart and demonstrated viral particles in interstitial cytopathic cells, most likely macrophages, but not cardiomyocytes or endothelial cells [90]. Direct cardiotoxic effects and presence of SARS‐CoV‐2 in the heart need to be confirmed in larger series.

As ACE2 is abundantly expressed by endothelial cells throughout the body, it loses its ability to prevent thrombosis upon cell entry of SARS‐CoV‐2 [13]. In human umbilical vein endothelial cell (HUVEC) cultures *in vitro*, ACE2 has been shown to have a role in protection of endothelial function and inhibition of the inflammatory response [91]. In experiments with spontaneously hypertensive rats, ACE2 activation reduced thrombus formation and platelet attachment to vessels, while these effects were reversed by inhibition of ACE2 [92]. Putatively, direct infection of endothelial cells by SARS‐CoV‐2 could result in systemic impaired microcirculatory function in different vascular beds. In fact, SARS‐CoV‐2 has recently been shown to directly infect engineered human blood vessel organoids *in vitro* [93]. The permissiveness of endothelial cells *in vivo* for SARS‐CoV‐2 was demonstrated in renal glomerular endothelial cells by electron microscopy [94]. However, since no immunohistochemistry or immune electron microscopy was performed, it remained difficult to distinguish between intracellular viral inclusions and normal subcellular organelles, as the latter may masquerade as viruses [95]. Furthermore, COVID‐19 was associated with endotheliitis in various organs such as the lung, liver, heart, kidney, and small bowel [94, 96]. This suggests that direct infection of endothelium and/or perivascular inflammation may result in endothelial dysfunction, tissue oedema, and a pro‐coagulant state culminating in microvascular pathology, in particular in patients with pre‐existing endothelial dysfunction.

### Thrombo‐embolic risk

COVID‐19 patients are at particular risk for developing coagulopathy reminiscent of disseminated intravascular coagulation (DIC) which was associated with mortality, possibly due to both venous and arterial thrombosis [97]. Arterial thrombosis includes ischaemia of the extremities, cerebral infarctions, and myocardial infarctions [98]. After initial reports of an increased rate of venous thromboembolism (VTE), including deep venous thrombosis (DVT) and pulmonary embolism (PE), a recent Dutch study demonstrated a VTE incidence of 27% and ~4% arterial thrombosis in COVID‐19 patients admitted to the ICU [99]. In this study, the vast majority (80%) of patients with VTE suffered from PE. PE could be an important factor in abrupt worsening of respiratory failure in patients with advanced COVID‐19 [100]. Furthermore, several autopsy studies showed thrombi in the pulmonary vessels, which can be proximal large emboli but are most frequently identified as microthrombi. This microvascular thrombosis is predominantly observed in an environment of marked inflammatory changes including mononuclear cell infiltrates, virally infected cells, and diffuse alveolar damage [88].

In clinical studies, strongly elevated levels of circulating biomarkers of endothelial activation have been reported [101]. Also, a clear picture of hypercoagulability is observed, with elevated D‐dimers being most strikingly elevated in patients with severe disease [40, 102, 103, 104, 105]. D‐dimer is a fibrin‐degradation product that develops after a blood clot is degraded by fibrinolysis. Moreover, D‐dimer levels at hospital admission predict a worse clinical outcome [6, 97]. Although D‐dimers are a biomarker for thrombosis, they are also known as strong acute‐phase reactants. However, high D‐dimer levels seem to persist in advanced COVID‐19 patients in whom inflammatory markers such IL‐6 have already decreased, stressing that their elevation is not solely secondary to systemic inflammation [6]. Furthermore, as COVID‐19 patients generally present with normal to slightly elevated platelet levels, strongly increased fibrinogen, and normal to only slightly prolonged prothrombin and activated partial thromboplastin time [106], thromboembolic events in these patients do not seem to be a result of a hypofibrinolytic consumptive diffuse intravasal coagulation as generally observed in sepsis [99].

Interestingly, strongly increased levels of antiphospholipid (anticardiolipin and anti‐β_2_‐glycoprotein I) antibodies have been reported in COVID‐19 patients with venous and arterial thromboembolisms, which is a feature of the antiphospholipid syndrome (APS) [102, 107]. Patients with systemic lupus frequently present with APS and limb ischaemia caused by vasculopathy. In a clinical study in systemic lupus, anti‐ACE2 antibodies were found to be elevated in almost every patient and correlated with the relative activity of serum ACE2 [108]. Furthermore, systemic lupus patients overexpress ACE2 as a result of hypomethylation, and their vascular complications respond very well to hydroxychloroquine treatment, being circumstantial evidence of a speculative link between ACE2 and vascular complications in COVID‐19 [109]. In summary, these observations underline that the hypercoagulable state in COVID‐19 may be of a systemic nature, and not limited to PE [110].

### Gastrointestinal involvement

Gastrointestinal (GI) symptoms are commonly observed in patients with COVID‐19. In a meta‐analysis of 4243 patients, pooled prevalence of gastrointestinal symptoms was 17.6% [111]. Moreover, viral RNA has been repeatedly detected in stool samples [112, 113]: in the aforementioned study, the pooled prevalence of positive samples was 48.1%. Commonly observed GI symptoms include anorexia, diarrhoea, vomiting, and abdominal pain [114]. In this study, diarrhoea as initial disease symptom was reported in 17% of patients, but seemingly no bloody diarrhoea. In addition, patients with digestive symptoms seemed to have a longer time from disease onset to hospital admission and presented with evidence of prolonged coagulation and elevated liver enzyme levels [114]. Theoretically, SARS‐CoV‐2 could directly invade the gastrointestinal epithelium via ACE2. In a single‐cell transcriptome study, ACE2 was found to be highly expressed in oesophageal upper and stratified epithelium, as well as in absorptive enterocytes derived from both the ileum and the colon [115]. In addition, ACE2 was co‐expressed with the TMPRSS2 prime protein in absorptive enterocytes and upper oesophageal epithelial cells. In our previous study from 2004, we found ACE2 to be expressed in enterocytes of all parts of the small intestine, including the duodenum, jejunum, and ileum, but not in colonic enterocytes [7]. More specifically, ACE2 was densely stained at the villous brush border, but also deeper into the intestinal wall, particularly in smooth muscle cells of the intestinal muscular layers, and in vascular smooth muscle cells and endothelium. Previously, proteomics analyses demonstrated that ACE2 protein is increased in IBD [116]. Furthermore, ACE2 activity and elevated angiotensin(1–7) concentrations were described in patients with IBD [117]. In that study, it was shown that Ang II and angiotensin(1–7) influence colonic myofibroblast proliferation and collagen secretion, and the use of ACE inhibitors (ACEIs) and angiotensin‐receptor blockers (ARBs) associated with improved disease outcome in IBD patients [118]. Until now, there is no evidence for increased susceptibility for COVID‐19 in patients with IBD. The implications of COVID‐19 for immunomodulation in IBD have recently been reviewed [119]. Previously, viral RNA in faeces could be detected after viral RNA in the respiratory tract became negative and evidence for gastrointestinal infection of SARS‐CoV‐2 was documented recently, i.e. infectious virus could be isolated from the stool [120, 121]. However, another recent study did not find evidence for the presence of infectious virus in RNA‐positive stool samples [122]. Altogether, these observations suggest that SARS‐CoV‐2 actively infects and replicates within the GI tract, implying a possible role for a faecal–oral viral transmission route.

### Liver involvement

Liver manifestations have also been reported in COVID‐19 patients. Biochemical signs of mild‐to‐moderate liver injury are frequently observed, including elevated liver function tests (AST, ALT, γ‐GT, and ALP), hypoalbuminaemia, prolonged prothrombin time, and increased CRP, LDH, and hyperferritinaemia, which may be reflective signs of acute‐phase inflammation [123]. Liver damage may be primarily attributed to direct viral infection causing hepatitis, but may also be interpreted as drug toxicity by administration of high‐dose antiviral medications, antibiotics or steroids [124]. ACE2 is expressed in the liver, mainly on cholangiocytes instead of hepatocytes, and it has been suggested that ACE2 might be upregulated by compensatory hepatocyte proliferation upon cholangiocyte injury [125]. To date, however, little is known about direct viral infection of the liver by SARS‐CoV‐2. One study on liver biopsy specimens showed moderate microvascular steatosis and mild lobular and portal activity, though it was unclear whether this was caused by SARS‐CoV‐2 infection or by drug toxicity [126]. Another study observed mild lobular infiltration by small lymphocytes, patchy necrosis, and centrilobular sinusoidal dilation [77]. Interestingly, a recent single‐cell transcriptomics study found high ACE2 expression on cholangiocytes, suggesting that SARS‐CoV‐2 may also lead to damage of intrahepatic bile ducts [127]. Taken together, one may hypothesise that hepatobiliary involvement in COVID‐19 primarily results from biliary infection, with secondary injury to hepatocytes.

### Renal involvement

Recent evidence points towards significant involvement of the kidney in COVID‐19. Whereas initial studies reported a relatively modest risk for acute kidney injury (AKI), subsequent studies reported an incidence rate up to 15% [128]. Occurrence of AKI in COVID‐19 patients is associated with higher disease severity in ICU‐admitted patients, and is an adverse prognostic sign for survival [129]. Small studies of COVID‐19 patients have reported signs of proteinuria and haematuria in about 40% of hospital‐admitted patients [130]. ACE2 expression has been confirmed on the brush border of proximal tubular cells and on podocytes, whereas glomerular endothelial and mesangial cells were weakly positive or negative for ACE2 [7]. In the previous SARS outbreak, renal involvement was a rare phenomenon, although, if present, AKI was often a fatal disease complication [131]. Further research provided evidence that this renal involvement, in the form of AKI, may be more attributed to processes behind multi‐organ failure rather than active viral replication of SARS viruses [131, 132]. For instance, CRS or cytokine storms have been reported as prior events leading to severe renal damage [133]. More recently, SARS‐CoV‐2 viral antigens have been detected in post‐mortem specimens, specifically in kidney tubules [134, 135]. In another recent study, histopathological analysis of post‐mortem findings revealed diffuse acute tubular injury (ATI) with loss of brush border integrity, non‐isometric vacuolar degeneration, and even necrosis, as well as prominent erythrocyte aggregates occluding the capillary lumina with resulting endothelial damage (Figure 4C,D) [136]. In line with the tissue distribution of ACE2 in the kidney, coronavirus‐like particles were identified in tubular epithelium and in podocytes. Based on these recent findings, it is suggested that SARS‐CoV‐2 directly targets the kidney parenchyma, especially the renal tubular epithelium and podocytes, with secondary endothelial injury that may induce AKI and lead to proteinuria and elevated serum creatinine levels in these patients. Moreover, SARS‐CoV‐2 infections seem to be more frequently associated with AKI compared with SARS‐CoV‐1 [130]. The increased binding affinity of SARS‐CoV‐2 to ACE2 may explain this phenomenon, as it would allow for greater renal infectivity.

### Skin involvement

In skin, ACE2 has been demonstrated in the basal epidermal layer and eccrine sweat glands [7]. However, reports on skin involvement started to emerge only recently. The extent and origin (reactive, direct viral damage, thrombosis, vasculitis) of skin involvement in COVID‐19, and the relation to severity of COVID‐19, remain to be established. Following two large COVID‐19 cohort descriptions only mentioning ‘skin rash’ without further details in a minority of patients [40, 137], several case reports have emerged reporting abnormalities ranging from erythematous rash, urticarial plaques, and purpura to chickenpox‐like vesicles, without information on histopathology [138, 139, 140, 141, 142, 143, 144, 145]. In addition, Recalcati reported skin alterations similar to the aforementioned case reports in 18 of 88 (20.4%) medication‐naive COVID‐19 patients [146]. Again, no histopathology was available. Very recently, one case report linked COVID‐19 to the occurrence of immune thrombocytopenic purpura [144]. Additionally, another study reported purpura and livedo racemosa in several severely affected COVID‐19 patients with small vessel thrombosis with co‐localization of complement and SARS‐CoV‐2 spike proteins on histopathology [147].This indicates direct viral infection of the small skin vessels. However, the diversity of skin features reported in COVID‐19 patients suggests other pathogenic mechanisms as well. In healthy skin, the layers above the ACE2‐expressing stratum basale, including the stratum corneum, likely provide a barrier to the virus. However, the clear expression of ACE2 in skin suggests that if SARS‐CoV‐2 gets the chance to reach its receptor there, for example through damaged skin, it may be able to render itself a *porte d’entrée* in keratinocytes. Given that SARS‐CoV‐1 was previously found in sweat [148], this raises the question of whether SARS‐CoV‐2 could be excreted in sweat, thereby adding to its transmission potential. In addition, it raises the question of whether SARS‐CoV‐2 is able to infect through binding to ACE2 in the eccrine sweat glands of palmar skin, where they are abundantly expressed.

### Placenta and pregnancy

Pregnancy is a unique physiological state in which a semi‐allogeneic fetus (and placenta) is accepted by the maternal immune system, whilst at the same time this system has to maintain the protective capacity for defence against pathogens. Due to the necessary adaptations in the immune system and a variety of physiological adaptations (e.g. increased oxygen consumption, mucosal oedema of the respiratory tract), pregnant women are generally characterised by increased susceptibility to respiratory pathogens, and consequently, severe pneumonia. Although there is no evidence that pregnant women are more susceptible to SARS‐CoV‐2 infection, they may be at increased risk of developing severe illness when contracting SARS‐CoV‐2 infection. Currently, there is limited evidence regarding the possibility of mother–fetal intrauterine vertical transmission in COVID‐19. Most descriptions of SARS‐CoV‐2‐infected pregnant women reported infections during the third trimester of pregnancy [149, 150, 151, 152]. However, uncertainty prevails about whether vertical transmission of COVID‐19 may occur in any phase of pregnancy [105, 106]. Placentas, amniotic fluid samples or newborns (directly after delivery) with positive RT‐PCR results have not been described, which means that there is no virological evidence of intrauterine infection at the maternal–fetal interface [149, 153, 154, 155]. Neonatal COVID‐19 has been reported, but infection could have occurred through other routes as there was no direct evidence for intrauterine vertical transmission [149, 151, 153, 156, 157]. In a small case series, fetal growth restriction (FGR) has been described in SARS‐CoV‐1‐positive women, but no details of the placental histopathological lesions were described [158]. There is one report that described seven placentas that were evaluated histopathologically after maternal infection with SARS‐CoV‐1. Placentas from infection in the first trimester were normal (*n* = 2). Increases in intervillous and subchorionic fibrin deposition were observed once delivered in the acute stage of infection (*n* = 3), which is possibly not SARS‐CoV‐specific, but rather related to disturbances in maternal placental blood flow due to hypoxic respiratory disease. Third‐trimester convalescent infection resulted in extensive fetal thrombotic vasculopathy (FTV) with sharply demarcated zones of avascular fibrotic villi resulting in FGR (*n* = 2). The aetiology of the FTV might be related to thrombotic tendency due to SARS‐CoV infection or placental hypoxia [159]. ACE2 could also play a role in this process. However, although placental ACE2 expression is found on both the fetal site (umbilical cord, placental villi in the syncytiotrophoblast, cytotrophoblast, vascular endothelium, and smooth muscle cells) and the maternal site (e.g. in the invading and intravascular trophoblast and decidual cells), regulation of placental ACE2 expression has not yet been described in relation to SARS‐CoV‐2 infection [160, 161].

### Neurological involvement

Neutropic potential of SARS‐CoV‐2 has been implicated in COVID‐19 [162]. Indeed, some patients presented with symptoms that could be attributed to neurological involvement, such as headache, confusion, anosmia, dysgeusia, nausea, and vomiting [163, 164]. Previous research showed that SARS‐CoV‐1 and MERS‐CoV infect the central nervous system with significant involvement of the brainstem [162]. It has been suggested that neuroinvasion of the brainstem may be at least partially responsible for respiratory symptoms in COVID‐19 patients, by compromising neurons within the respiratory centres and chemosensitive neural cells involved in respiratory and cardiovascular regulation [165]. ACE2 may play a role in SARS‐CoV‐2 neuroinvasion, as it is expressed in the brain on neurons and glial cells, particularly in the brainstem and cardiovascular regulatory areas, including the nucleus tractus solitarius, paraventricular nucleus, and the rostral ventrolateral medulla [166, 167]. In addition, ACE2 is expressed in the cerebral vascular endothelium, which could lead to endothelial damage subsequently leading to viral access to the brain [168, 169]. In an experimental animal study, it was demonstrated that SARS‐CoV‐1 enters the brain primarily via the olfactory bulb, followed by transneuronal spread of the virus [170]. This could explain the underlying pathophysiology of COVID‐19‐associated anosmia. However, detailed neurological investigation of COVID‐19 autopsies should further clarify the occurrence and underlying neurological pathology characteristic of SARS‐CoV‐2 infection.

## Pathogenesis and treatment options for COVID‐19

Initially, SARS‐CoV‐2 may either pass through the mucous membranes in the upper respiratory tract, primarily the nasal and pharyngeal epithelia, or directly enter the lower respiratory tract and infect bronchial and alveolar epithelial cells [11]. The main symptoms of respiratory infection are fever and cough. In this initial phase, the virus can enter the peripheral bloodstream via the lungs and this may result in viraemia [171]. The virus may then proceed to affect other organs expressing ACE2, such as the heart and blood vessels, the kidneys, and the GI tract. However, the GI tract may also be directly infected by the oral route. Patients with an increased risk of developing severe disease may experience severe pulmonary involvement resulting in systemic inflammation [6]. The massive inflammatory process at that time results in a severe cytokine storm also affecting other organs in the body. This seemingly occurs in line with other blood‐derived viruses entering organs via ACE2 on activated endothelium causing, for example, renal or GI problems. In the vasculature, this coincides with red blood cell aggregation and thrombosis. The clinical phase progresses from the initial viraemia to an acute phase (pneumonia), followed by either recovery or severe disease (including ARDS, AKI, and eventually multi‐organ failure) requiring ICU admission [172]. The distinction would depend on patient comorbidity, obesity‐induced pre‐existent inflammation, immune function, and ACE/ACE2 balance in already affected organs. Each phase demands its own treatment regimen ranging from virus entry and replication inhibition in the initial phase to anti‐inflammatory and anti‐thrombotic medication at later stages. In the following paragraphs, we aim to highlight some of the most commonly advocated treatment strategies being explored to combat COVID‐19.

## Antiviral drugs

### Hydroxychloroquine and chloroquine

Hydroxychloroquine (HCQ) and chloroquine (CQ) are two widely used antimalarial, antiviral, and anti‐rheumatic drugs. Recently, *in vitro* results and small clinical studies emerged that demonstrated antiviral activity of these drugs against SARS‐CoV‐2 infection [173, 174, 175, 176, 177, 178]. Beneficial effects were presumed to arise from blockage of viral host cell entry by increasing endosomal pH and interference with glycosylation of ACE2 [179]. Two studies from France reported that HCQ could lead to viral load reduction within 6 days, especially when combined with azithromycin. However, these studies were impaired by several methodological constraints [180]. Similarly, two Chinese trials were performed: one study reported no significant difference in nasopharyngeal viral carriage between HCQ treatment and standard supportive care, whereas the other study demonstrated a shorter clinical recovery time for patients receiving HCQ compared with placebo [177, 178]. For the latter study, however, it was not possible to extrapolate to critically ill patients, which is crucially important because this subgroup of patients may be of particularly increased risk of serious adverse effects upon treatment with HCQ/CQ, such as ventricular arrhythmias, hepatic failure, and cardiac toxicity [180, 181]. Indeed, recent studies reported concerns about potential safety hazards as higher dosages were associated with higher mortality and excessive QT interval prolongation, especially when taken concurrently with azithromycin and oseltamivir [182, 183]. Another large observational study indicated that HCQ/CQ may not help in critically ill patients as its administration was not associated with either a significantly lowered or an increased risk of a composite endpoint of intubation or death [184]. Thus, currently available data on HCQ/CQ treatment for COVID‐19 are inconclusive, but appear far from promising. Therefore, upcoming prospective randomised clinical trials will have to determine if treatment with HCQ/CQ would be a reasonable therapeutic strategy for COVID‐19 patients and what would be the most suitable timing within the disease course to initiate treatment.

### Remdesivir and lopinavir/ritonavir

Remdesivir, an RNA polymerase inhibitor, was demonstrated to be effective against SARS‐CoV‐1 and MERS‐CoV. For instance, remdesivir improved disease outcome and reduced viral load in SARS‐CoV‐1‐infected mice [185]. In 53 hospitalised patients with COVID‐19, improvement of clinical status was observed in 36 patients after receiving at least one dose of remdesivir [186]. Furthermore, a recently conducted randomised controlled trial evaluated the role of lopinavir and ritonavir in 199 COVID‐19 patients: 99 were treated with lopinavir/ritonavir, while 100 received standard treatment [187]. The authors concluded that patients treated with lopinavir/ritonavir did not demonstrate any significant improvement in hazard ratio for earlier clinical improvement or reduction in mortality at 28 days. In contrast to the primary outcome, patients treated with lopinavir/ritonavir demonstrated clinical improvement 1 day earlier than the control group and were discharged 5 days earlier from the ICU. Although large clinical trials investigating the therapeutic effect of these antiviral therapies in COVID‐19 are lacking, it can be hypothesised that the available studies possibly included patients with severe disease alone, and therefore future studies may consider evaluating the role of these antiviral drugs earlier in the course of COVID‐19 [187].

## RAAS inhibitors

The worldwide growth of SARS‐CoV‐2 infections has raised serious concerns about the widespread use of antihypertensive drugs, i.e. ACEIs and ARBs, which are also used in the treatment of cardiovascular diseases, chronic kidney disease, and diabetes mellitus [188]. Discussions emerged about whether these drugs may exert beneficial or deleterious effects in COVID‐19. Many opinion papers have been published recently that predominantly state that there is no scientific evidence to change the prescription of ACEIs or ARBs for the management of hypertension in the context of preventing or treating SARS‐CoV‐2 infection. The use of ACEIs and ARBs as risk factors for developing or aggravating COVID‐19 has been suggested because of their capacity to upregulate ACE2 [189, 190, 191]. However, others have advocated beneficial and protective effects of these drugs in the development of COVID‐19 [15, 188].

In some animal studies, ACEIs or ARBs increase ACE2 levels, whilst other studies failed to demonstrate such shifts in ACE2 [13, 20, 192, 193, 194, 195, 196, 197, 198, 199], although shifts in ACE/ACE2 balance were noted [20]. Therefore, it remains relevant to question whether RAAS blockers actually increase susceptibility to SARS‐CoV‐2 infection by increasing ACE2. ACE2 is protective against severe lung injury in animal models and ACE2 blockade or genetic *Ace2*‐knockouts result in extensive lung damage and decreased survival after respiratory syncytial virus infection [200]. Similarly, AT_1_R blockade by losartan attenuates lung injury in mice administered with the spike glycoprotein of SARS‐CoV‐1 [25]. Although few human studies have been performed investigating the potential effects of RAAS therapy on ACE2 expression and/or activity, it was recently reported that ACEIs and ARBs did not increase plasma ACE2 concentrations [45]. Similarly, others reported no clear direct effects of ACEIs on ACE2 activity [as evaluated by angiotensin(1–7) levels] [201, 202].

Several hypotheses exist about how increased tissue ACE2 expression may be protective rather than harmful during SARS‐CoV‐2 infection [203]. For example, increased ACE2 expression may lead to enhanced sequestration of SARS‐CoV‐2, but does not imply automatic activation of further downstream processes essential for viral entry, such as involvement of TMPRSS2, which is required for spike glycoprotein priming, or ADAM metallopeptidase 17 (ADAM17), which is required for cleavage of the ACE2 ectodomain resulting in increased ACE2 shedding. Furthermore, ARBs lead to competition with Ang II for AT_1_R, resulting in increased Ang II to be processed by ACE2. This increases Ang(1–7) levels, which results in vasodilating and anti‐fibrotic effects, providing crucial protection during coronavirus infections [25]. Furthermore, increased binding of ACE2 to circulating Ang II could induce a conformational change resulting in less favourable binding of SARS‐CoV‐2 to its receptor and decreased internalisation of the virus when bound to ACE2 [203, 204].

We previously observed a positive shift in plasma Ang(1–7)/Ang II balance in favour of the beneficial Ang(1–7) peptide, particularly in circumstances of low sodium intake [19]. Importantly, however, plasma ACE2 levels may be less indicative of the risk of SARS‐CoV‐2 infection or membrane‐bound ACE2 activity, as ACE2 shedding by ADAM17 appears to be regulated separately [205]. Interestingly, however, plasma ACE2 concentrations appear to be higher in older men with heart failure, independent of RAAS inhibition [45].

Clinical trials investigating the potential (side‐) effects and safety of ACEIs and ARBs on ACE2 expression and activity in COVID‐19 are ongoing. From a clinical perspective, it may be preferable to await these results instead of discontinuing RAAS inhibitors, which may lead to clinical derangement especially in patients at high risk for COVID‐19 [206]. Since currently available evidence indicates that ACEIs and ARBs significantly reduce mortality in cardiovascular disease, reduce progression of CKD, and are crucial in the treatment of heart failure and hypertension, most clinicians tend to maintain these regimens for their patients, regardless of SARS‐CoV‐2 [188].

## Biological response modifiers

Immunomodulating drugs or biological response modifiers alter the host immune system by interacting with a specific target crucial for disease pathogenesis [207]. Many of these compounds enrich the therapeutic armamentarium of several malignancies, autoimmune disorders, transplantation rejection, as well as infectious diseases. Especially since vaccine development is time‐consuming and antiviral drugs may have a limited therapeutic window, targeted immunomodulators are attractive alternatives. Furthermore, these therapies may be crucial to control the hyperactivation of host inflammatory responses and the ‘cytokine storm’ as has been described for COVID‐19 [208]. However, caution should be taken towards this therapeutic strategy as it will remain challenging to target immune system components without compromising the host defence mechanisms necessary to fight SARS‐CoV‐2 infection. In this respect, targeting specific or limited effector mechanisms (e.g. production of pro‐inflammatory cytokines or reactive oxygen species) should be preferred over blockage of more proximal immune targets (e.g. pattern recognition receptors) that play a more significant role in regulating host immune defence [208].

### Anti‐cytokine therapy

The current hypothesis is that a cytokine storm can induce or further aggravate SARS‐CoV‐2 infection, and thereby suggests that blocking cytokine pathways could attenuate the disease course. Among these, interleukin‐6 (IL‐6) is thought to play a prominent role. IL‐6 is a cytokine with both anti‐ and pro‐inflammatory effects. It can be produced by almost all stromal and immune system cells (monocytes, lymphocytes, macrophages, endothelial cells, mast cells, dendritic cells) and is believed to play a central role in the development of a cytokine storm [209, 210]. In line with this reasoning, anti‐IL6R therapy is a potential therapeutic option in COVID‐19. Currently available humanised monoclonal antibodies against the IL‐6 receptor (tocilizumab and sarilumab) are being tested in COVID‐19. A small study demonstrated that tocilizumab ameliorated the increased CRP in all 15 patients, which is a direct effect of its pharmacological action. Moreover, in critically ill patients with elevated IL‐6 levels, repeated doses of tocilizumab could be beneficial. However, objective clinical endpoints were not reported [211]. Although others have shown comparable results, data on the use of tocilizumab are still preliminary and larger randomised controlled trials are needed [212, 213, 214]. Whether anti‐IL6R therapy should be started early in the course of the disease or restricted to patients with signs of a cytokine storm is still of debate [209]. In addition, other cytokines such as IL‐1, IFN‐γ, and TNF‐α are abundantly present in the cytokine storm, and the potential of blocking their pathways with appropriate biologicals is currently being investigated [215].

### Janus kinase (JAK) inhibitors

Inhibition of the JAK–STAT signalling pathway has also been suggested as a potential targeted therapy for COVID‐19 and several clinical trials are ongoing [216, 217]. Inhibitors blocking JAK2, such as fedratinib, have been suggested to block viral entry and combat the Th17 component of the host inflammatory cytokine storm, without altering interferon signalling [218]. SARS‐CoV‐2 enters host cells via ACE2‐mediated endocytosis, which is controlled by upstream regulators including AP2‐associated protein kinase 1 (AAK1) and cyclin G‐associated kinase (GAK). One of several high‐affinity inhibitors of these regulators is the JAK inhibitor baricitinib, which may limit viral host cell entry and intracellular assembly of viral particles through disrupting AAK1 and GAK. Baricitinib may be of particular value during the hyperinflammatory phase, in which high levels of cytokines occur that signal through the JAK–STAT pathway. However, the optimal time to administer cytokine inhibitors still needs to be determined and results from the aforementioned clinical trials should be awaited.

## Resveratrol

The association between obesity and the progression to hypoxic respiratory failure in patients with COVID‐19, requiring mechanical ventilation, has led to the assumption that leptin and adipokines may play a key role in this subpopulation of SARS‐CoV‐2‐infected patients. Resveratrol, an antioxidant and food supplement, has been suggested to be of potential therapeutic value because of a triple action. First, in some studies, resveratrol reduces leptin levels [219]. Second, resveratrol could suppress Ang II, which might reduce inflammation [220]. Third, antioxidant effects in the lung may reduce oxidative stress‐induced lung damage [221]. This food supplement is safe in its use (up to 2–3 g per day) and should be studied in COVID‐19 patients as an additive to other treatments.

## Anticoagulant treatment

As a result of the increased risk of thrombotic events in COVID‐19, guidelines currently advocate liberal use of prophylactic systemic anticoagulation [222]. The International Society on Thrombosis and Haemostasis recently recommended that all hospitalised COVID‐19 patients, even those not admitted to the ICU, should receive prophylactic‐dose low‐molecular‐weight heparin (LMWH) unless they have contraindications (active bleeding and platelet count less than 25 × 10^9^ per L) [223]. However, a recent study showed that despite adequate treatment with prophylactic low‐dose LMWH, COVID‐19 patients admitted to the ICU were still at a substantial risk for PE [99]. This has made the Dutch Federation of Internists decide to recommend a double dose of LMWH in ICU patients with COVID‐19, when bleeding risk allows this strategy [224]. Other guidelines advocated prophylactic systemic anticoagulation with unfractionated heparin rather than LMWH [225], which may be needed in high dosages because of heparin resistance [226]. However, it is unlikely that anticoagulant treatment has a direct disease‐modifying effect and it should be stressed that the initial viral load, as well as the systemic inflammatory response, needs to be attenuated since these are the driving forces for VTE in COVID‐19 [227]. Future studies are warranted to determine the most suitable approach for thrombosis prophylaxis in COVID‐19.

## Concluding remarks and future perspectives

ACE2 is widely distributed throughout human tissues and a myriad of factors have been implicated in influencing its expression and functional activity. Genetics, demographic characteristics, lifestyle, varying comorbidities, and medication usage are all considered to have an impact on ACE2 expression and activity. With the ongoing rapid spread of novel scientific findings about ACE2 and its role in COVID‐19 pathophysiology, it is crucial to maintain integration of available pathological and molecular evidence to establish the definite role of these potential modulating factors.

Unravelling the pathologic basis of COVID‐19 is essential for our understanding of the pathophysiology of the disease. Unsurprisingly, severe pathological findings are mainly observed in specific target organs of SARS‐CoV‐2, such as the lungs and kidneys. In severe cases, this may lead to ARDS and multi‐organ failure not directly related to ACE2 expression and activity. This review focused on the role of widespread ACE2 tissue expression, which may become a reasonable therapeutic target together with its effector pathways, for example through implementation of recombinant human ACE2 (rhACE2) therapy or by targeting bradykinin metabolism in the lungs. However, it will also be important to focus on additional mechanisms that may be involved in cellular infection and may regulate the interaction between SARS‐CoV‐2 and ACE2.

Future studies featuring higher numbers of patients are warranted to reliably assess potential differences in ACE2 expression, activity, and regulation under a variety of physiological circumstances, such as present or lacking interaction with co‐receptor or co‐activating molecules, as well as in the context of commonly observed underlying conditions, including cardiovascular disease, hypertension, diabetes, obesity, smoking, and respiratory disease. In particular, pathological studies of larger series of autopsy findings, probably in human and non‐human primate models alike, are required to more accurately determine the relative contribution of each pre‐existent comorbidity and to discriminate between specific SARS‐CoV‐2‐associated pathology and superimposed pathological changes. Furthermore, the development of appropriate animal and *in vitro* models could help us to learn more about the SARS‐CoV‐2 infection process itself and, most importantly, the disease progression pattern observed in humans. In any case, it is indisputable that devoting scientific efforts to analyse aspects of ACE2 in relation to COVID‐19 pathophysiology is paramount to fuel the development and augmentation of future therapeutic strategies.

The current COVID‐19 pandemic is a major challenge for public health and clinical medicine. For public health, reduction or prevention of virus transmission as well as reduction of predisposing lifestyle factors needs to be implemented. For clinical management in the foreseeable future, we should strive to adopt a *personalised medicine* approach aimed to provide individually tailored treatment in patients affected by COVID‐19. As highlighted in this review, this should take into account individual patient differences in mutual ACE2–SARS‐CoV‐2 interactions with their consequences for COVID‐19 pathophysiology.

To achieve this, it is of cardinal importance to carefully register the individual patient phenotype and to integrate this with diagnostic (e.g. laboratory and imaging results) and therapeutic information (e.g. drug toxicity and side‐effect profiles). Mainly because of low patient numbers in individual studies, currently ongoing trials are challenged to take into account between‐subject differences or cohort heterogeneity, which may be considered likely to explain most of the variation in disease outcome. However, detailed phenotypical stratification of individual patients during their disease course will provide us with the necessary input for sophisticated clinical algorithms to be used for predictive modelling. Consequently, these will allow us to identify rational therapeutic strategies tailored to a patient’s clinical status. As such, we would distance ourselves from the ‘one size fits all’ approach, and it would enable us to identify novel avenues for therapeutic modulation for COVID‐19 and future viral diseases.

**Abbreviations**
AAK1: AP2‐associated protein kinase 1
ACE2: angiotensin‐converting enzyme 2
ACEi: ACE inhibitor
AKI: acute kidney injury
Ang I: angiotensin I
Ang II: angiotensin II
APS: antiphospholipid syndrome
ARB: angiotensin‐receptor blocker
ARDS: acute respiratory distress syndrome
AST: aspartate aminotransferase
AT_1_R: angiotensin‐II type 1 receptor
BMI: body‐mass index
CQ: chloroquine
CRP: C‐reactive protein
CRS: cytokine release syndrome
cTnI: cardiac troponin I
cTnT: cardiac troponin T
DVT: deep venous thrombosis
eQTL: expression quantitative trait loci
FGR: fetal growth restriction
FTV: fetal thrombotic vasculopathy
GAK: cyclin G‐associated kinase
GI: gastrointestinal
HCQ: hydroxychloroquine
IBD: inflammatory bowel disease
ICU: intensive care unit
IMID: immune‐mediated inflammatory disease
LDH: lactate dehydrogenase
LMWH: low‐molecular‐weight heparin
PE: pulmonary embolism
RAAS: renin–angiotensin–aldosterone system
S‐protein: spike protein
VTE: venous thromboembolism

## Author contributions statement

HvG, ARB, ADMEO, GJN, AAV, and PHJvdV were involved in conceptualisation. ARB, AEA, and HvG designed the review outline. ARB, AEA, and HvG wrote the first draft. DJM, MCB, SJG, JLH, GD, and WT wrote sections of the review. All the authors contributed to manuscript revision and approved the submitted final version.
